# The Role of Parents and Children in Meal Selection and Consumption in Quick Service Restaurants

**DOI:** 10.3390/nu12030735

**Published:** 2020-03-11

**Authors:** Juliana F.W. Cohen, Eric B. Rimm, Kirsten K. Davison, Sean B. Cash, Kyle McInnis, Christina D. Economos

**Affiliations:** 1 Department of Public Health and Nutrition, Merrimack College, 315 Turnpike Street, North Andover, MA 01845, USA; 2 Department of Nutrition, Harvard T.H. Chan School of Public Health, 677 Huntington Ave, Boston, MA 02115, USA; erimm@hsph.harvard.edu; 3 Channing Division of Network Medicine, Department of Medicine, Brigham and Women’s Hospital and Harvard Medical School, 75 Francis Street, Boston, MA 02115, USA; 4 Boston College School of Social Work, McGuinn Hall 115, 140 Commonwealth Ave, Chestnut Hill, MA 02467, USA; kirsten.davison@bc.edu; 5 Friedman School of Nutrition Science and Policy, Tufts University, 150 Harrison Ave, Boston, MA 02111, USA; sean.cash@tufts.edu (S.B.C.); christina.economos@tufts.edu (C.D.E.); 6 Merrimack College, 315 Turnpike Street, North Andover, MA 01845, USA; mcinnisk@merrimack.edu

**Keywords:** quick service restaurants, child diet, plate-waste, fast food, feasibility

## Abstract

Children regularly consume foods from quick service restaurants (QSRs) in the United States, but little is known about how ordering decisions are made and the impact on selection and consumption. A total of *n* = 218 parents dining with a child (ages 4–16 years) inside a participating QSR completed interviews and demographic surveys and provided their child’s leftover foods at the end of the meal. Children’s meal consumption was measured using plate-waste methodology. The majority of children selected their meal without parental involvement (80%) and decided what to order prior to entering the QSR (63%). Using mixed-model analysis of variance, children selected and consumed significantly fewer calories and less total fat and sodium when a parent ordered the meal compared with when the child ordered the meal alone. There were no significant differences in selection or consumption when a parent and child ordered the meal together. Approximately one-third of the children consumed foods that were shared. In conclusion, because children primarily select foods without parental involvement and prior to entering QSRs, innovative strategies are needed to influence ordering decisions inside QSRs toward healthier options. Additionally, because food is frequently shared, policies that only focus on children’s menus may not be as effective in impacting children’s dietary intake.

## 1. Introduction

Over the past 30 years, spending at quick service restaurants (QSRs) in the United States has risen from roughly $6 billion to $110 billion, and children’s percentage of daily calories attributed to QSR food consumption has increased from 2–13% [1,2]. QSR foods are currently the second-largest source of empty calories from solid fats and added sugars in children’s diets, with roughly 33% of children consuming foods from QSRS on any given day [3,4,5,6]. This can have important health implications for children, as they tend to consume significantly more calories, saturated fat, sodium, sugar, and sugar-sweetened beverages (SSBs), as well as fewer fruits and vegetables on days that they have foods from QSRs [5,7]. There is also evidence for a positive association between consuming foods from QSRs and body mass index (BMI) among children [8,9,10,11]. However, QSRs are increasingly providing healthier options, which also have the potential to lead to substantial reductions in the caloric content of the meals ordered for children [12,13,14,15,16]. Because QSR food consumption is both frequent and normative for many families, research is needed to better understand how ordering decisions are made by children and parents in QSRs in order to develop interventions and policies to nudge consumers towards the healthier options available [5].

Children’s food consumption is strongly influenced by their parents, but children have played a greater role in decision-making regarding food purchases over the past few decades [17,18,19]. Children’s autonomy typically increases as they develop cognitively, with increased abilities to process information and make choices, while research also suggests societal shifts in the role of children within families may also play a role, with participatory models replacing more authoritative ones [19]. Previous research examining parent-child decision-making in food establishments has primarily been conducted in supermarkets and has found that children strongly influence buying decisions [20,21,22]. One study conducted in full-service restaurants found that children frequently order meals without parental involvement [23]. However, the role of both children and parents in ordering decisions and the impact on the foods ordered and consumed by children in QSRs is currently unknown. The aims of this study were to evaluate parent-child decision-making to better understand how and where decisions were made regarding which foods to select in QSRs, the influence on consumption, and how this differed by the child’s age.

## 2. Materials and Methods

Data collection took place in four stand-alone QSRs from one national restaurant chain. The research team collaborated with the QSR chain to select locations in New England with an emphasis on socioeconomically and racially diverse settings reflective of the QSR chain nationally. All subjects gave their informed consent for inclusion before they participated in the study. The study was conducted in accordance with the Declaration of Helsinki, and the protocol was approved in May 2016 by the Merrimack College Institutional Review Board (Project identification code RB-FY17-18-159).

### 2.1. Participants and Recruitment

Participants were parents or legal guardians (herein referred to as “parents”) who were at least 18 years old and purchasing food for a child between the ages of 4–16 years for consumption inside of the QSR. A total of *n* = 218 parents were recruited (63% of the eligible parents approached). Parents provided verbal consent and were provided with a participant information document.

Data were collected from January 2018 through January 2019 between 11 a.m. and 8 p.m on Saturdays and Sundays. All Saturdays and Sundays throughout the year were included in the study, with the exception of *n* = 9 weekends due to data collection location transitions, and were spread throughout the year. All adults with children in the participating QSRs were approached to participate in the study by trained research assistants (RAs) while waiting in line after either deciding on meal selections (to prevent influencing interactions or meal choices) or immediately after placing their order. As part of the recruitment process, adults confirmed that they had not previously participated in this study. At the end of the meal, the RAs interviewed participating parents using a script with open-ended questions and prompts that had previously been pilot tested among parents in QSRs. The questions focused on how ordering decisions were made inside of QSRs that day and in general, including who decided on the child’s meal and what factors influenced those decisions. Example questions included, “Who made the decision [to order the food for your child]?” and “Did you decide for your child or did your child decide for him/herself?” The RAs also probed for additional information on how and where decisions were made. Based on these questions, the role of parents and children in ordering decisions were coded as “parent alone,” “child alone,” or “child and parent together.” Participants also filled out a survey with demographic information for both themselves and their child. For families that were in the QSR with multiple children who met the age requirements, the child with the closest birthday to the data collection date was included in the study to ensure random selection. 

### 2.2. Plate-Waste Methodology

To assess children’s selection and consumption in the QSRs, plate-waste measurements in the QSRs were conducted using standard methodology previously used in QSR settings [24]. At the end of the meal, parents provided all remaining foods, condiments, and containers and/or wrappers from the child’s meal. Parents also described all of the foods ordered for the child, including any modifications to the menu items. When parents requested to bring leftover foods home, these foods were weighed onsite (*n* = 4 food items). All other foods were brought back to a lab in sealed bags for plate-waste measurements. Foods were weighed on food scales (OXO 1130800, OXO Company, New York, NY, USA) in grams.

For each unique food item leftover by participants, two additional samples were ordered, weighed, and averaged by RAs to obtain a stable estimate of the pre-consumption weight for the food (i.e., two samples of regular sized fries were obtained as the baseline weight for any participant ordering regular fries). Percent consumption was calculated using the formula:

$$\frac{Average\;preweight\;of\;the\;two\;food\;samples-Postweight\;of\;the\;participant^{\prime }s\;food}{Average\;preweight\;of\;the\;two\;food\;samples}\times 100$$

Parents were also asked to provide detailed information on the quantities of foods shared when applicable. When a parent provided a specific number for a shared food item (i.e., six fries), this quantity was weighed from the pre-weight sample to estimate the percent consumed. To calculate nutrients consumed by the child, the percentages from the plate waste were multiplied by the nutrients provided by the QSRs on their websites. This was calculated for each food item and summed for the overall meal to determine the total nutrients consumed.

### 2.3. Analyses

Descriptive statistics were used to characterize the study participants, the distribution of parent and/or child ordering decisions (i.e., “child alone,” “parent alone,” or “child and parent together”), where decisions occurred (prior to entering or inside of the QSR), and the frequency of sharing meals. The association between parent and/or child ordering decisions and nutrients selected and consumed were examined using mixed-model analysis of variance with restaurant location as a random effect. All models were adjusting for child age, child sex, and child ethnicity. Age was categorized as “child” or “adolescent,” with the “child” category further subdivided based on typical literacy rates, thus creating three age groups: Preliterate children (ages 4–6), literate children (ages 7–12), and adolescents (ages 13–16). Because of the proximity to other QSR chains, many families who ate in the participating QSRs brought in food from other QSRs, and these food items were also included in the study. Therefore, the QSR chain was also adjusted in the analyses. Estimates when comparing ordering decisions were calculated using least squares means regression. Parent education was not a significant confounder and therefore not controlled for in the final analyses.

## 3. Results

The characteristics of the participating parent-child pairs are presented in Table 1. Approximately one-third of participants (35%) were fathers, 49% were mothers, and 16% identified as other. Overall, 35% of participants had a college degree. On average, children were 9.6 years old (range 4-16 years) and half were female. Half of participants identified their ethnicity as Hispanic, and, for race, almost half (46%) identified as white. Overall, 24% of participants reported that their families consumed meals at QSRs at least once a week, 40% reported a few times a month, 17% reported once a month, and 19% reported a few times per year. 

**Figure 1:**
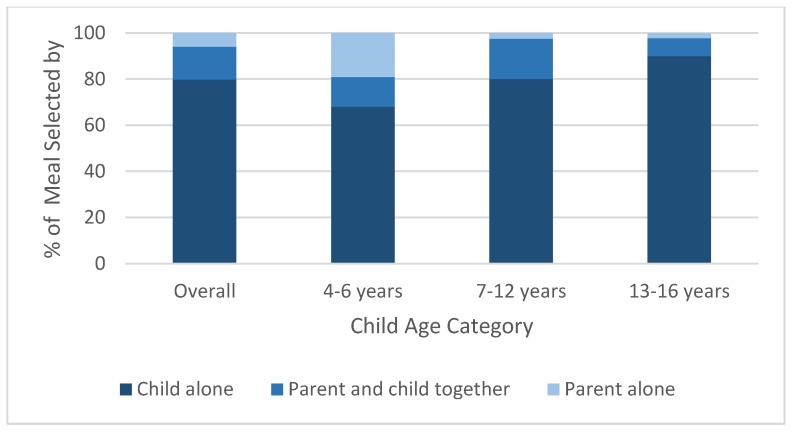
Ordering decisions by child age category inside of *n* = 4 quick service restaurants (QSRs) in New England.

When deciding what to order, 80% of children decided without any parental involvement, 6% of parents decided without child involvement, and 14% of parents decided with their children, although this varied by the age of the child (Figure 1). Compared with parents in QSRs with older children, parents with children who were six years old or younger played a greater role in ordering decisions, although the majority of young children ordered without any parental involvement. Whereas 68% of parents reported that they played no role in their child’s order, 19% of parents decided alone and 13% decided with their child. Among parents with 7–12-year-old children, 80% did not play a role in their child’s ordering decision, 3% decided without the child’s involvement, and 17% decided with their child. Among the oldest children (ages 13–16 years), 90% of parents played no role in their child’s decision, 2% of parents decided for their child, and 8% of parents involved their child. 

The majority of parents (63%) reported that the child knew what he or she was going to order prior to entering the QSR. Approximately 30% of parents reported that the price of the food items inside of the QSR influenced ordering decisions, including which or how many items could be ordered (e.g., several parents stated that their child could order any menu items as long as their total meal was approximately $5 or less).

When examining the meals ordered for children, nearly all included at least one entrée (96%) and a beverage (87%), 34% included a side dish, and 14% included a dessert (Table 2). There were no significant differences in the selection of entrées, beverages, side dishes, or desserts by parent-child decision-making (parent alone, child alone, or parent and child together). Overall, the meals as ordered had, on average, 855 calories, 33.2 g of total fat, 10.0 g of saturated fat, 1585 mg of sodium, 7.6 g of fiber, and 58.1 g of sugar.

**Figure 2:**
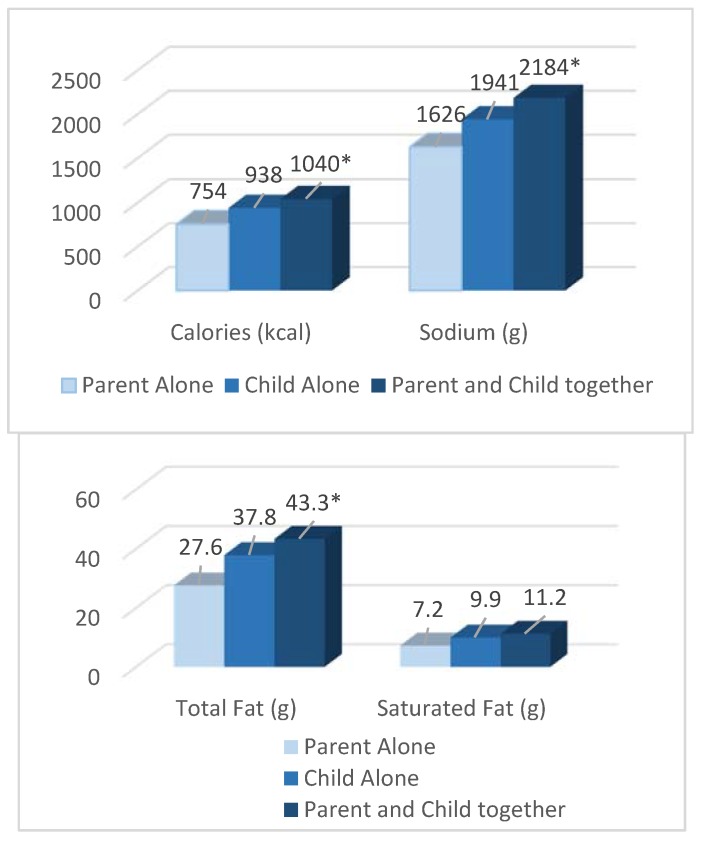
Selection of nutrients from the overall meal by who made the ordering decision (parent alone, child alone, or child and parent together). * Significantly different (*p* < 0.05) by who made the ordering decision (parent alone (reference group), child alone, or child and parent together) using mixed-model analysis of variance with restaurant location as a random effect and adjusting for QSR chain, child age, child sex, child ethnicity, parent sex. Values were calculated using least squares mean regression.

Among the nutrients in the meals ordered by parent-child decision-making, significant differences were observed (Figure 2). When a parent and child ordered together, the overall meal (entrée, beverage, side, and dessert combined) had, on average, 285 more kcal compared with when a parent alone ordered for a child (1040 kcal vs. 75 4kcal; *p* = 0.03), although there were no significant differences compared with when the child ordered alone (938 kcal; *p* = 0.10). There was also significantly more total fat in the meals ordered by a child and parent together compared with parents alone (43.3 g vs. 27.6 g; *p* = 0.007), and with meals ordered by the child alone (37.8 g; *p* = 0.04). Additionally, sodium was higher in meals ordered by parents and children together compared with meals ordered by just the parent (2184 mg vs. 1626 mg; *p* = 0.04), but there were no significant differences compared with children who ordered alone (1940 mg; *p* = 0.21). Last, there was a trend toward ordering meals with more saturated fat when the parent and child decided together compared with meals ordered by the parent alone (11.2 g vs. 7.2 g; *p* = 0.05). There were no significant differences in the amount of sugar ordered by who made the ordering decisions.

When evaluating the individual meal components ordered, entrées had, on average, 218 calories more when a parent and child ordered together compared with when a parent ordered without the child’s input (640 kcal vs. 422 kcal; *p* = 0.04), although there were no significant differences compared with when the child ordered alone (583 kcal; *p* = 0.10). Similarly, there was significantly more total fat in the entrées ordered by a child and parent together compared with entrées ordered by parents alone (34.2 g vs. 21.1 g; *p* = 0.02), but there was only a nonsignificant trend toward selecting entrées with more total fat compared with entrées ordered by the child alone (29.8 g; *p* = 0.07). There was also a trend toward ordering higher sodium entrées by a parent and child together compared with when the parent decided alone (1655 mg vs. 1137 mg; *p* = 0.05), but no significant differences compared with entrées ordered by the child alone (1477 mg; *p* = 0.16). For all of the other meal components (i.e., beverages, side dishes, or desserts), there were no differences in nutrient levels by who made the ordering decision.

**Figure 3:**
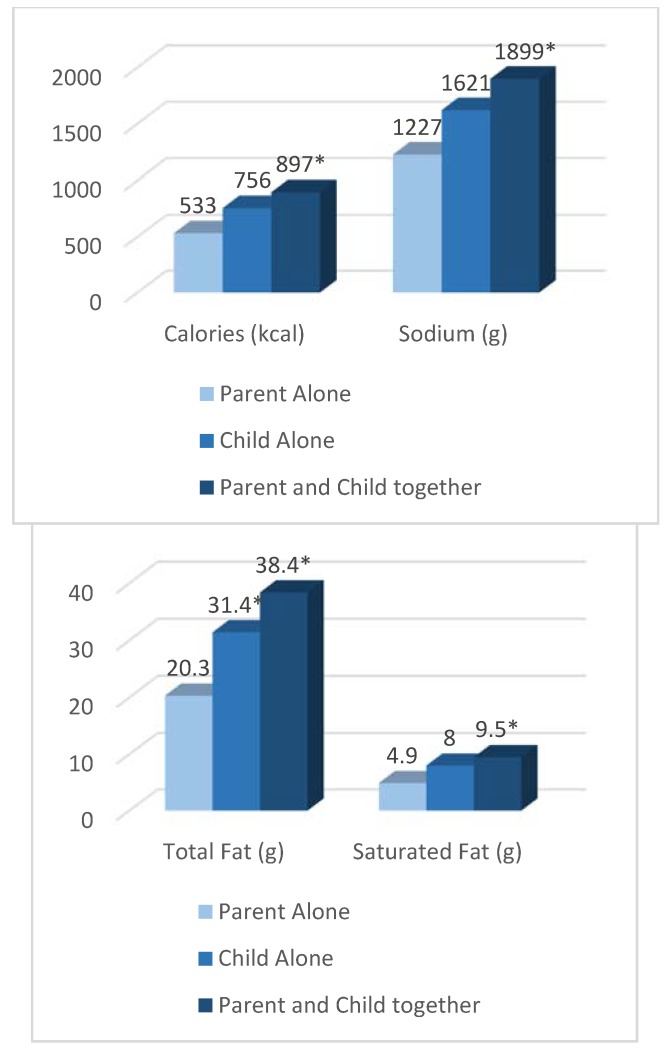
Consumption of nutrients from the overall meal by who made the ordering decision (parent alone, child alone, or child and parent together). * Significantly different (*p* < 0.05) by who made the ordering decision (parent alone (reference group), child alone, or child and parent together) using mixed-model analysis of variance with restaurant location as a random effect and adjusting for QSR chain, child age, child sex, child ethnicity, parent sex. Values were calculated using least squares mean regression.

When examining meal consumption, nearly one-third of the children (31%) consumed foods that were purchased either for another family member or for the family to share. Children ate significantly more of their meal overall when a parent and child ordered together compared with meals ordered by the parent alone (85.2% vs. 70.2%; *p* = 0.04) or compared with when the child ordered alone (83.0%; *p* = 0.04 (Table 3)). As a result, children consumed significantly more calories when the parent and child ordered together compared with when the parent alone ordered the meal (897 kcal vs. 533 kcal; *p* = 0.007), although there was only a trend toward more consumption compared with when the child ordered alone (756 kcal; *p* = 0.05 (Figure 3)). Similarly, children consumed more saturated fat when parents and children ordered together compared with parents alone (9.5 g vs. 4.9 g; *p* = 0.02), and a trend was observed only when compared with children who ordered alone (8.0 g; *p* = 0.07). When examining total fat, children consumed more total fat when parents and children ordered together compared with parents alone (38.4 g vs. 20.3 g; *p* = 0.002), as well as children who ordered alone (31.4 g; *p* = 0.03). Last, children also consumed more sodium when they ordered with a parent compared with when their parents ordered for them (1899 mg vs. 1227 mg; *p* = 0.02), but there were no significant differences compared with children who ordered alone (1621 mg; *p* = 0.13). When examining individual meal components, no significant differences in the percent or nutrients consumed were observed. Sharing foods was also frequent among the participants. 

## 4. Discussion

This study found that the majority of children, including young children, ordered food at a QSR without parental involvement and decided what to order prior to entering the QSRs. Additionally, parental involvement in ordering decisions with a child was not protective. It was only when a parent ordered the child’s meal without the child’s involvement that the overall meal had significantly fewer calories, and less total fat and sodium, although differences in total fat were observed only when compared with meals that the child ordered alone. Similarly, children consumed significantly fewer calories, as well as less total fat, saturated fat, and sodium when the meal was selected by the parent alone compared with meals selected by the child and parent. 

Similar to a previous study by Elbel and colleagues examining adolescents in QSRs [25], this study found that joint parent-child food decisions did not impact the nutrients in the meal ordered compared with when the child ordered without parental involvement. In fact, the present study found that compared with orders made by the parent, orders tended to have more calories when a parent and child ordered together, although no significant differences were observed when the child ordered alone. However, it is possible that with the relatively small number of adults deciding for their child, this study was underpowered to detect smaller changes in the caloric content of the meals ordered by an adult versus a child alone.

This study also found that children frequently ordered without parental involvement, decided before entering the QSRs, and consumed foods shared among family members. The results of this study were similar to those found by Castro and colleagues examining ordering decision of families with children ages 3–14 in San Diego full-service restaurants, which found that 60% of children knew what they were going to order prior to entering the restaurant and 34% shared foods [23]. Given the large amount of sharing observed in restaurants, this suggest that policies only impacting children’s meals may be limited. Not surprisingly, that study also observed child autonomy when placing a meal order increased with older children, although parents played a much larger role in that study across all age groups compared with the present study. It is possible that these differences are due to differing characteristics of people who tend to dine in QSRs compared with full-service restaurants, although future research is necessary to replicate these findings.

The high percentage of children deciding on their meals prior to entering the QSRs may in part explain why research examining the current calorie labeling policies in QSRs have found minimal impacts on the meals selected and consumed by children, although these policies can still lead to potentially meaningful changes in diets due to product reformulation [25,26,27,28]. Future research should examine parent-child decision-making the first time a child goes to a QSR to better understand and potentially influence ordering decisions before habits are formed. Additionally, research is needed to better understand how parent feeding styles and the frequency of fast food consumption are associated with ordering decisions. Studies should also examine innovative behavioral economic strategies within QSRs aimed at modifying prior decisions to encourage changing children’s decisions to the healthier options available, such as through the interactive kiosks increasingly available in QSRs. Modifications to menu boards, such as enhanced food descriptors or images and verbal prompts, may also encourage the selection of healthier foods. Given the importance of meal price, healthier, lower-cost combo meal options may also effectively lead to healthier selection and consumption in QSRs.

This study had several limitations. First, data were collected in only four QSRs in New England. However, these sites were selected in collaboration with the QSR chain for their potential generalizability to the chain nationally. It is also possible that participation in this study influenced parent-child decision-making, selection, or consumption. However, because research assistants discussed the study only with the parents after orders had been decided and did not directly state that consumption would be assessed, this likely minimized any influence. Last, data were only collected on weekends, although this enabled both lunch and dinner data to be collected among a diverse population. Strengths of this study included detailed data from interviews and plate-waste measurements among a socioeconomically and racially diverse population with both mothers and fathers in a ‘real-world’ setting.

## 5. Conclusions

This study is one of the first to examine parent-child decision-making when ordering inside of a restaurant setting. This study found that children predominantly selected their foods prior to entering QSRs and without parental involvement, which is important for QSRs, researchers, and policymakers to take into consideration when designing future policies, interventions, or legislation. Additionally, parental involvement with their children in the ordering decision was not protective and resulted in more calorie-dense meals. Innovative strategies, such as verbal prompts, menu boards, or electronic kiosks that highlight healthier options may help nudge children at the point of sales. Last, because food is frequently shared in QSRs, policies that only focus on children’s menus may not be as effective in impacting children’s dietary intake.

## Figures and Tables

**Table 1:** Participant characteristics of parents and children ordering meals in QSRs.

**Parent/Guardian (*n* = 218)**	
	%
Female	61
Relationship to child	
Mother	49
Father	35
Other	16
Education *	
Some high school or less	13
High school	21
Some College	28
College	35
**Child (*n* = 218)**	
	mean (range)
Age, yrs	9.6 (4–16)
	%
Sex, % Female	50
Ethnicity, % Hispanic/Latino	50
Race	
Asian	7
White/Caucasian	46
Black/African American	3
More than one race	41
Frequency of Fast Food Consumption	
≥ Once a week	24
A few times a month	40
Once a month	17
A few times a year	19
Role of Parent and/or Child in Ordering Decisions (%) **	
Child Alone	80
Parent Alone	6
Child and Parent Together	14

* *n* = 6 parents did not respond. ** Inside of the QSR on the day of data collection. quick service restaurants (QSRs).

**Table 2:** Average selection of meal components and nutrients for children’s meals ordered in QSRs.

	% Selecting	Calories ± SD (kcal (Range))	Total Fat ± SD (g (Range))	% Calories from Total Fat ± SD (% (Range))	Saturated Fat ± SD (g (Range))	% Calories from Saturated Fat ± SD (% (range))	Sodium ± SD (mg (Range))	Fiber ± SD (g (Range))	Sugar ± SD (g (Range))	% Calories from Sugar ± SD (% (Range))
Entrée ^1^	95.9	546 ± 286 * (160–2200)	27.3 ± 14.2 * (7.0–90.0)	45.4 ± 6.8 (13.3–59.0)	8.8 ± 5.2 (0.5–36.0)	15.0 ± 5.3 (0.8–23.5)	1287 ± 726 (280–4760)	6.5 ± 3.8 (0.0–23.0)	3.1 ± 2.2 (0.0–13.0)	2.3 ± 1.2 (0.0–5.2)
Beverage	87.2	218 ± 107 (0–420)	0 ± 0 (0–0)	0 ± 0 (0–0)	0 ± 0 (0–0)	0 ± 0 (0–0)	93 ± 102 (0–390)	0 ± 0 (0–0)	58.6 ± 28.6 (0.0–110.0)	88.7 ± 31.4 (0.0–100.0)
Side Dish^2^	33.5	293 ± 101 (90–470)	15.4 ± 6.9 (2.0–30.0)	45.8 ± 9.0 (15.0–72.0)	3.5 ± 2.7 (0.0–9.5)	10.6 ± 8.0 (0.0–42.0)	638 ± 244 (190–1330)	3.2 ± 1.6 (0.0–9.0)	3.2 ± 3.8 (0.0–11.0)	4.1 ± 4.8 (0.0–24.0)
Dessert	13.8	188 ± 51 (160–310)	8.3 ± 3.7 (6.0–18.0)	38.9 ± 7.6 (31.8–52.3)	2.0 ± 2.1(0.0–4.5)	9.6 ± 10.3 (0.0–22.5)	167 ± 62 (85–260)	1.0 ± 0.0 (1.0–1.0)	14.6 ± 2.1 (13.0–20.0)	31.8 ± 4.8 (21.4–37.5)
Overall	–	855 ± 360 * (210–2480)	33.2 ± 16.5 * (8.0–92.0)	34.7 ± 8.7 (13.1–56.7)	10.0 ± 5.4 (1.5–36.0)	10.8 ± 4.2 (1.7–20.0)	1585 ± 840 * (335–5360)	7.6 ± 3.6 (1.0–23.0)	58.1 ± 33.4 (0.0–120.0)	27.8 ± 17.4 (0.0–75.6)

* Significantly different (*p* < 0.05) by who made the ordering decision (parent alone (reference group), child alone, or child and parent together) using mixed-model analysis of variance with restaurant location as a random effect and adjusting for QSR chain, child age, child sex, child ethnicity, parent sex. ^1^ Nutrients were for all entrées combined (*n* = 3 meals included two entrées). ^2^ Nutrients were for all side dishes combined (*n* = 4 meals included two side dishes).

**Table 3:** Average consumption of meal components and nutrients for children’s meals in QSRs.

	Consumption ± SD (% (Range))	Calories ± SD (kcal (Range))	Total Fat ± SD (g (Range))	% Calories from Total Fat ± SD (% (Range))	Saturated Fat ± SD (g (Range))	% Calories from Saturated Fat ± SD (% (Range))	Sodium ± SD (mg (Range))	Fiber ± SD (g (Range))	Sugar ± SD (g (Range))	% Calories from Sugar ± SD (% (Range))
Entrée	90.0 ± 18.8 (14.0–100.0)	497 ± 291 (43–90)	24.7 ± 14.1 (2.0–90.0)	45.2 ± 6.9 (13.3–60.4)	8.0 ± 5.0 (0.5–36.0)	15.0 ± 5.3 (0.8–23.5)	1161 ± 723 (78–4760)	6.0 ± 3.7 (0.0–23.0)	2.9 ± 2.2 (0.0–12.0)	2.4 ± 1.2 (0.0–5.2)
Beverage	71.0 ± 30.4 (2.0–100.0)	168 ± 83 (3.2–420)	0 ± 0 (0–0)	0 ± 0 (0–0)	0 ± 0 (0–0)	0 ± 0 (0–0)	67.8 ± 71.1 (0.8–390)	0 ± 0 (0–0)	45.4 ± 22.3 (0.9–110)	99.8 ± 1.0 (92.3–100.0)
Side Dish	82.1 ± 21.3 (10.0–100.0)	229 ± 90 (41–470)	11.9 ± 5.8 (1.5–30.0)	45.6 ± 9.0 (15.0–72.0)	2.7 ± 2.2 (0.0–8.5)	10.7 ± 8.5 (0.0–42.0)	492 ± 210 (92–918)	2.4 ± 1.2 (0.0–5.0)	2.6 ± 3.3 (0.0–13.0)	4.5 ± 6.1 (0.0–30.6)
Dessert	85.5 ± 28.9 (20.0–100.0)	159 ± 67 (32–310)	6.9 ± 3.7 (1.2–18.0)	38.2 ± 7.4 (31.8–52.3)	1.6 ± 1.9 (0.0–4.5)	8.8± 10.3 (0.0–22.5)	146 ± 75 (17–260)	0.9 ± 0.3 (0.2–1.0)	12.2 ± 4.4 (2.6–20.0)	31.6 ± 4.8 (21.4–37.5)
Overall	82.5 ± 18.3 * (2.8–100.0)	699 ± 361 * (83–2480)	28.4 ± 15.9 * (0.0–90.0)	36.2 ± 9.7 * (0.0–60.4)	8.7 ± 5.2 * (0.0–36.0)	11.4 ± 4.7 (0.0–21.5)	1343 ± 814 * (18–5100)	6.6 ± 3.7 (0.0–23.0)	39.6 ± 28.3 (0.0–116.1)	24.4 ± 19.0 * (0.0–100.0)

* Significantly different (*p* < 0.05) by who made the ordering decision (parent alone (reference group), child alone, or child and parent together) using mixed-model analysis of variance with restaurant location as a random effect and adjusting for QSR chain, child age, child sex, child ethnicity, parent sex, and the role of price in ordering decision.
